# Extra Supplement—The Nursing Mirror

**Published:** 1889-03-23

**Authors:** 


### The Nursing Mirror.

All Contributions for this Supplement should be addressed "The Nursing Mirror," care of The Editor, 38, Old Jewry, E.C.

### En Passant.

LADY NURSES.—A discussion lately took place at Manchester Infirmary about lady nurses. Mr. Simpson and Mr. Alfred Neild approved of ladies as nurses, while Alderman King disapproved. Finally, it was decided that ladies might apply to be taken on as probationers at Manchester Infirmary if they liked. This was very kind. There are not many infirmaries left where the shortsighted policy of excluding ladies is still maintained.

ESTABLISHING DISTRICT NURSES.—Anyone who wants to set about starting a district nurse should secure the annual report of Ealing Cottage Hospital, which has just been published. During the past year the attention of the committee has been especially engaged on the establishment of a branch dispensary at Ealing Green, in connection with the hospital. Most of the subscribers are aware that, in addition to the hospital for the reception of in-patients, there is a provident dispensary carried on at the hospital. This branch of the charity has been of very great service, and there are now six hundred members belonging to it. In establishing the branch dispensary two nurses were also provided to attend on the members in their own homes, and the committee describe how this was done, and then express their satisfaction with the nurses, and with the matron, Miss Reid.

A FEW WORDS TO MIDWIVES.—An inquest was held last week on the death of Alice Evans, wife of a lighterman, who, in her confinement, was attended by a midwife. Puerperal fever set in, and the midwife called in the attendance of a doctor's assistant, knowing him not to be a qualified medical man. The patient died—through the gross ignorance of the unqualified assistant, the jury said; and the midwife was removed from the Court in a fainting condition. Here is one more proof that we should all hasten the day when midwives should be registered by Act of Parliament. It must be remembered that a midwife differs from a nurse, in that she attends cases solely on her own responsibility, and does not, except in abnormal labour, work under a medical man. She is, in fact, a practitioner in one branch of medicine, and as such should always be qualified and registered. We are glad the Midwives' Institute has maintained the status of its members, by insisting on this view. All midwives should read Dr. Galabin's address on "Modern Progress in Obstetrics," reported in last week's *British Medical Journal*.

DISTRICT NURSES.—Sir Rutherford Alcock has written a long letter to the *St. James's Gazette*, pointing out the fallacies about lady nurses which a morning paper has been spreading. He says: "The improvement that has taken place in the last quarter of a century in the training and education of nurses, the apportionment of their duties, and the provision made for their accommodation and health within and without the walls of the hospitals, leaves so little to be desired that no vocation is more eagerly sought after by women of the educated classes or more constantly followed when once entered upon." Sir Rutherford considers that hospital nurses are not overworked, and that all pity should be kept for district nurses, who "must, indeed, be prepared in the homes of the poor to do a great deal of menial and repulsive work, with none to assist; and, in the absence of all appliances, to put a sick room in 'nursing order,' as they are required to do for their first duty. This labour has to be undertaken again and again, often in the same day; while she has to move in all weathers from house to house within her district, traversing several miles, it may be, before the day's labour comes to an end."

DEATH OF MISS WHATELY.—Those who are interested in the nursing work at Cairo, so much spoken of at present, will deeply regret the death of Miss Mary Whately, head and founder of the English Mission Schools there. She was the daughter of Archbishop Whately, and went first to Cairo in 1861 for her health. She was much struck with the necessity for philanthropic work in Egypt, and wrote several books on the subject. She stirred up the interest of England, and obtained the money to start schools, a dispensary, and other charities. She was much interested in the new nursing home, and her familiarity with Cairo peculiarities would have made her a valuable friend to nurses settling there.

TROUBLES AT STROUD.—The following two resolutions passed by the Sanitary Committee of Stroud Road Hospital, Gloucestershire, speak for themselves: "That this committee being satisfied that certain complaints against Mrs. Harper, now acting as nurse at the hospital, were well founded, instruct the Town Clerk to write informing her that her services are not longer required." Resolved on February 22nd: "That in the opinion of this committee some alteration is desirable in the arrangements for taking charge of the hospital, Stroud Road, and the nursing of patients therein; and that the council be recommended to empower this committee to deal with the matter as they may think best."

NURSES AS STEWARDESSES.—We have now got thirty names on our register of nurses willing to act as stewardesses, and shall enter no more names until such time as these first thirty shall have had their chance of taking a trial voyage. The nurses we have registered are all certificated, thoroughly-trained nurses, and many of them have had experience of the sea, and know what they are volunteering for. Here is one who has crossed the Atlantic six times, and who, besides being a general nurse, has a certificate from the Lying-in Hospital; here is another, a woman of thirty-five, trained in one of the best schools, and possessing the diploma of the Obstetrical Society. Another has trained at Kimberley, another in New Zealand. One is a matron in an asylum, another a Sister in the Midlands. The shipping companies will indeed be difficult to please if they cannot select suitable stewardesses from such a register. We must again warn our readers not to expect this new movement to be started in a few days. We are working on steadily with the plan, for we are sure it is a good one, but time is needed to perfect all things.

SHORT ITEMS.—The Islington Guardians have informed the *Lancet* that their infirmary nurses are trained. Yes; by whom? We doubt if one untrained woman can train others.—At their meeting on March 12th the York County Hospital Committee stated that, Miss Lowe having resigned the matronship, Miss Forrest, of Guy's Hospital, was appointed in her place. During the year the work of training paying probationers has been increased, the hospital now training them for Newcastle Nurses' Home, the Victoria Hospital for Children, Chelsea, Kent Nursing Home, and the Leicester Home, in addition to the York Home.—The Bristol Children's Hospital Committee, with a view to establishing a training school of nursing last spring, engaged two hospital-trained, experienced ladies, and further advertised for probationers who desired to go through a two years' course. The new arrangement is now in full working order. The staff consists of the matron, two nursing sisters—one of whom superintends the work on each corridor—eight nurses, and eight probationers, including those on night duty and those in the fever annexe.

### Insurance for Women.

A Lecture at the Somerville.

A very interesting paper on this subject was read by Mrs. Symes Thompson at the Somerville Club on Tuesday, the 12th inst., to a large and interested audience, which included officials from several well-known and successful insurance companies. Mr. Burrage, actuary to the Equitable Life Insurance Company—the oldest life office in the kingdom—occupied the chair. The lecturer gave a short history of insurance in general. An American preacher once declared Joseph to have been the founder of insurance, when he stored up a proportion of the corn grown in Egypt in the prosperous years as a provision for the time of famine. This is certainly the principle of insurance of all sorts—laying up something in the day of prosperity for the possible needs of the future. In England insurance on ships was the first to be established in the time of good Queen Bess, by her minister, Sir Thomas Gresham. Insurance against fire followed later, receiving a great impetus from the losses entailed by the Great Fire of London. Then came life insurance, now the most important branch of insurance business.

The lecturer gave several instances of the value of this provision for the future, by examples both of the misery that results when death occurs suddenly and nothing of the kind has been secured, and the almost dazzling possibilities of increase on the sum assured for by means of bonuses. As especially applicable to the case of women, who in many instances are more concerned to provide for their own old age than for the future of others, Mrs. Symes Thompson recommended endowment insurances. By these, the sum assured in case of death at any age, was handed over to the insurer at the age of fifty or sixty, according to the agreement made on taking out the policy, with all the bonus additions. Thus a man who at the age of twenty insured for £1,000 would receive £2,000 at the age of sixty, the sum doubling itself in that time, although his yearly premiums, if added together, would not amount to the original £1,000 he bargained for. Surely this is good interest on invested money; especially when the investment is made in small sums, such as it would be difficult to place advantageously in any other safe way.

Of course, women rarely earn such incomes as will permit them to insure for such a sum as £1,000; the yearly premium, however moderate in comparison with the sum to be paid, being beyond what they can afford; but nearly all can lay aside a few shillings a month, or a quarter. It would secure them something for old age, and probably, with bonus additions, that something would be a great deal more than was counted on. It may be necessary—for all women are not familiar with financial phrases—to explain what a bonus is. Every five or seven years the insurance company takes stock—counts up the funds in its possession. If it finds that there is more than enough to satisfy all claims, it divides the surplus among its policy holders, who then, as was said, get more than they bargained for; and, of course, the longer they postpone their claim the more they get.

In the discussion which followed, one gentleman spoke also of the value of surrender values. That is, if through unforeseen circumstances an insurer is unable to keep up his policy, the company will buy it from him, and give him for it almost the whole value of his premiums. Surrender values increase in a remarkable way with the age of the policy. If the insurer does not want to sell his insurance outright, he can, as Dr. Symes Thompson pointed out, exchange it for a paid-up policy of smaller amount. There was also some mention of those friendly societies which take small sums weekly, and are therefore most suitable for the working classes. In the Prudential Office, the assets of which amount to millions, the money is mostly collected in weekly twopences from the working classes. Some of these societies, however, are far from sound, and care must be taken to choose a good one. The practice o parents insuring their children was also spoken of—that is, insuring a sum to be paid to the child during its life; not those burial or "coffin" insurances, as Mr. Burrage called them, by which the parent profits by the child's death. Mrs. Charles, whose experience of the poor is large and varied, also condemned these.

In reply to a question from one of the audience about the National Pension Fund for Nurses, both Dr. and Mrs. Symes Thompson expressed their approval of the scheme, and their sympathy with it—the former pointing out that the nurses were following the example of their fellow-workers in the field of disease, the doctors, who had a similar fund of their own. Such funds, which often receive large sums in legacies and donations, are, of course, able to pay larger interest on money invested in their policies than ordinary insurance offices. Mr. Burrage also said: "*The Nurses' Fund will do good work; it is well officered, and seems to be admirably managed.*" Such testimony, from a gentleman whose impartiality and knowledge are unimpeachable, ought to settle the last doubt of the most timorous.

Women, as a rule, are quite ignorant of the value and even the meaning of insurance; but a clear, thoughtful and practical lecture, such as Mrs. Symes Thompson's, ought to do much to clear the cobwebs out of their brains. It would be well if Mrs. Symes Thompson could be persuaded to deliver it again to some other audience of working women.

### Nursing Journals and Amateur Editors.

(*From a Correspondent.*)

I desire to call the attention of your readers to the following paragraphs about the National Pension Fund, which appear in a contemporary: "We are placing the estimate very low when we suggest that a Nurse is very exceptionally fortunate if she is not invalided, *on the average*, for two weeks in each year. Now we find from one of our correspondent's letters that 6s. 8d. per month will secure her 8s. a week from the Fund if invalided. If each then received Sick Pay only for two weeks in every year, on the same scale, they would cost the Fund £432 a year; while their Premiums, invested at, say, 5 per cent., would only produce £108. And we must assert our belief that in practice the actual loss would be far greater than this, for many reasons. For example, a Nurse might poison her hand. Now she would go about and do her work as well as she could—perhaps have an abscess opened, and poulticed meanwhile. But with a vision of Sick Pay before her, she would ask for, and at once receive, a medical certificate; and she would draw her allowance for three weeks or longer and enjoy perfect rest, to the great advantage of her health. Or she might severely sprain her ankle. Now she would be hobbling about in ten days. With Sick Pay she would rest for at least a fortnight. What we wish to draw attention to, is the simple fact that Sick Pay for Nurses on their scale spells only one word, and that is —Ruin. It will doubtless be asked why we should, as representatives of Nurses, warn the Council, and not rather quietly encourage all our readers to seize the opportunity of aggrandising themselves." Here I will stop: I do not wish to quote one word more of this journal than is necessary. So long as it attacked only the Pension Fund I did not care; the managers of the Fund are business men, and know what they are about; but now this journal attacks nurses as a body, I wish to defend my fellow-workers. The first statement quoted above, that a nurse is laid up, on an average, two weeks a-year, needs proving. From the *Contemporary Review* I learn that the average amount of illness to an ordinary being is four days a-year. The difference between four days and fourteen days is too great to be believed unless supported by figures. The second statement I dispute is that nurses will make the most of petty ailments in order to secure sick pay. Such an imputation in a paper supposed to be written for our perusal is a disgrace alike to nursing and to journalism. Who are they who insinuate such low motives? for Shakespeare spoke truth in saying, "Suspicion haunts the guilty mind." Surely there are few even of the general public who will not feel the utmost indignation at such a slur cast on the noble band of nurses. The third statement I would call attention to is that your contemporary hesitates to urge its readers to aggrandise themselves at the expense of the Fund because their good feelings lead them to prefer to warn the council into what a financial quagmire they are wandering. The word "aggrandise" is, I suppose, meant for "enrich," but I do not cavil at mere ignorance; I would simply point out that in former numbers this same journal has said: "We do not consider the Fund to offer to nurses the advantages which they can obtain elsewhere for the same investment," and, "In matters of rates and conditions the National Pension Fund for Nurses cannot in any way favourably compare with the same value for the nurses' investments as could several established insurance societies." To warn the council their lavishness is ruinous, and to advise the nurses to seek better investments elsewhere, is transparent nonsense.

For my own sake and for that of my fellow-nurses I wish to show how little credence can be given to your contemporary, which accuses us of inclination to sham ill. In its forty-fifth number this journal, which has not yet run a year, says—"We have incurred much blame because of our studied silence with respect to the so-called Pension Fund for Nurses." The "studied silence" consists in devoting nine editorials, ten articles, and numerous letters and paragraphs to the Fund; in fact, there is scarcely a number of the journal in which the Fund is not mentioned with abuse. In its thirty-ninth number your contemporary stated: "Hitherto there has never been an organ in the English Press devoted wholly and solely to nurses' interests." The fact is that *Nursing Notes* was the first journal established for nurses, and has been carried on solely in their interests for nearly two years. A tree must be known by its fruits, and a journal by the fairness and justness of its comments, and the excellence and originality of its articles. There cannot be two opinions about a journal which largely consists of articles which have already appeared in other papers. One column anent a benevolent fund has appeared thirty times, and last week the same paragraph appeared twice in one page. Also, in the last number appeared an article on "Amateur Nursing," by a very amateur author. She was shocked at the grievous bodily harm which might befall the patient of an amateur nurse; why did she not remember the more grievous mental wrong she did to the victims who tried to struggle through her amateur article? But I am afraid I trespass too far on your valuable space; in conclusion I would merely ask your readers what they think of a journal which can offer such stupendously stupid lines as the following, as suitable literary matter for the perusal of nurses?—

Victoria took the gift,
And said, "What shall I do
With this most loyal offering?
Make it a *blessing* too!

"First, I will think of *him*—
My husband, loving, true,
Whose presence was my sun of life;
And then I think of you,

"I will provide the strong,
Kind arms to nurse the poor;
To make the bed and keep the house;
*So* I will spend My store!"

### An Ancient House of Mercy.

### CHRIST'S HOSPITAL, SHERBURN.

LAST week we gave an account of a religious community of modern times, and now we have received a most interesting history of a religious community of the olden days. The Master of Christ's Hospital, Sherburn, has written a little pamphlet telling the story of the foundation over which he presides. Some miles from Durham, in a secluded valley, is a considerable pile of buildings, commonly known as Sherburn Hospital. It was built in the twelfth century by the Bishop of Durham, for the reception of lepers of both sexes. The inmates numbered 65; for leprosy, introduced into England by the Crusaders, who contracted the disease in the East, was terribly common in England at that time; and, owing to the unhealthy—not to say dirty—habits of our ancestors, the contagion spread with great rapidity. There were two chapels connected with the hospital, one for the men and one for the women, but on great festivals High Mass was celebrated in the presence of the lepers of both sexes, who came in procession, headed by their respective Prior and Prioress. At this early date in England leprosy was not only regarded as incurable, but beyond alleviation, and no thought was taken to lengthen the lives of those who dwelt in Sherburn Hospital. They were plentifully fed with flesh and fish and eggs and vegetables, and fire and candles and many comforts were provided for all so long as they lived; but there seems to be no mention of medicines, or any treatment for the disease. But there was treatment for the souls of the lepers, and this of the strictest kind—midnight mass and matins were regularly performed, and a perpetual lamp was kept burning before the altar. During Lent and Advent the brethren and the sisters were required to receive corporal discipline in their respective chapels, and at all times a disobedient brother or sister might be chastised by the Prior and Prioress, or relegated to solitary confinement and bread-and-water, or even expelled for ever from the hospital.

The natural revulsion from such a state of affairs in religious houses took place at Sherburn as elsewhere; where there had been too great strictness there was shortly too great laxness, and during the dark middle ages the revenues of the hospital were turned to private uses, and its orders became a dead letter. Through the period of the Civil War, down to 1735, the hospital was in a state of constant tribulation and change. Bishop Chandler did his best to remedy a few of the abuses, but in 1854 the Charity Commission put forward an authoritative claim to somewhat direct the revenues.

Under a totally new scheme, which gave the management into the hands of fifteen governors, Christ's Hospital, Sherburn, bloomed forth once more as a House of Mercy. The salaries of all the officials were fixed, the number of brethren reduced to thirty; and £12,000 was put apart for building a large infirmary, to form one side of the quadrangle of the hospital buildings. All mention of the sisters vanishes as the pamphlet turns to modern times, but surely the nurses in the infirmary might maintain in dress, or habits, or title, some remnant of ancient days to remind new-comers how old is the foundation of the hospital? The infirmary holds thirty patients, fifteen of each sex, and in 1880 a dispensary was built at a cost of £2,000, which is attended by about 20,000 out-patients annually.

Such is the story Mr. Mitton tells of Christ's Hospital, Sherburn: a story full of interest, and full of thought for those who like to contrast the past and the present, to try and trace the steady progress of all things, and to learn the lessons which every true history teaches.

### Everybody's Opinion.

*[Correspondence on all subjects is invited. No communications can be entertained if the name and address of the correspondent is not given, or unless one side of the paper only be written on.]*

NURSES' SAVINGS FUND.

"Handclasp" sends a long letter, from which we take the following: On reading the numerous opinions about The National Pension Fund, I am strongly reminded of two doggrel lines I once heard:—

"They all agreed to disagree:
A most united familee."

Now, as long as this is the case, and there is no combined effort on the part of the nurses to help themselves, success in any one undertaking cannot be looked for. Nursing matters at the present moment seem rather in a bad way: the "Combination of Private Nurses" seems to have come to a dead lock, The National Pension Fund has all it can do to scrape together the required thousand members; The British Nurses' Association, in spite of a great deal of high falutin, has not advanced one iota further in its registration for nurses. In whatever direction one looks, failure stares one in the face, and all for the want of united effort. I was very pleased to see a letter dealing with the National Pension Fund, by an "Army Sister." When I wrote my paper, to which she so courteously alludes, it was in the hope nurses far more capable than myself would join in the discussion. Every opportunity should be given to nurses to speak of and understand the Pension Fund; it is of vital interest not only to us, but to every nurse who shall follow in our footsteps. Matrons, who are for the most part well-educated ladies, should take up the matter, and call a general meeting of their nurses, and explain clearly all about it, giving them every opportunity to ask questions, from the head Sister to the youngest probationer, at the same time guarding against bringing undue influence to bear on either side. All nurses having fully grasped the subject might then be asked for their votes for and against. It seems to me there is far too little active interest shown by nurses themselves. I should be grateful to have a little more information from an "Army Sister." May I ask what difference there is between this plan and the one adopted by the Post Office Savings Bank, except the bonnuses to depositors? Money is now at such low interest—5 per cent. is almost out of the question—and in arguing on matters of this sort the maximum of loss must be thought of, and the minimum of gain recognised. Where small savings are invested it should be a *sine qua non* that safety is the first consideration, and if an "Army Sister's" plan varies so little from that in vogue at the Post Office Savings Bank, would it not be just as well to place the money in there, which we all know has the Government for its security? When starting a fresh concern, say, a savings bank for nurses, it is generally in numbers that its safety and paying properties would have to rely upon, and it would be difficult to prognosticate, after all we have read, whether 10,000 or 15,000 nurses would combine in such an undertaking? How would an "Army Sister" regulate the distribution of bonuses? Would they be divided equally to the matron and probationer alike, to the one who deposits a £1 to the other's 1s.? Or would they be given to nurses according to pressing necessity? If so, it might be more advantageous for some to deposit only the 1s. Or would a large deposit ensure a large bonus? The great difference between an "Army Sister's" plan and the National Pension Fund is that in the former depositors always retain control over the principal, which many people think an advantage. [This is not the case. Members of the Pension Fund on the returnable scale can now use it as a bank, and withdraw their principal at any time before the pension begins to be paid.] One can in this case live on the interest during life, and will away the principal at death, or use it oneself under necessity. Of course, the old difficulty starts to the fore, viz., the impossibility of nurses, with their present small earnings, laying aside such a sum as would enable them to live on the interest. The committees, the boards of management, etc., should bestir themselves heart and soul to increase our salaries, and probably at less expense eventually to their pockets? We should then be in a position to welcome such a national fund as the one which has been started. I cannot and do not agree with the insolent tone adopted by some nurses and others, as regards this great and kind effort to help us. We nurses should do our best and look at the matter each for herself, in a clear, impartial manner. I can honestly state I am influenced by nobody. The future—such a melancholy future to some of us—may seem dim and far off in our younger days, but it is sure to come, relentlessly, silently, swiftly. Are we to meet it like dismantled ships—anchorless, rudderless, at the world's mercy?

LADY NURSES.

A. E. S. writes: May I say a word on the vexed question of "Lady Nurses"? How many ladies, I wonder, take up the work for the love of it? I for one did *not*, I had to earn my living, but I think I do my work as well as the romantic ones who gush over "Patients and Wards." Training was hard work, particularly to one who up to thirty years of age had been her own mistress, but it makes a great difference when your future bread depends on your training. I consider no work menial, and can clean taps and "do up fires" better than the probationers I now train. All women should know how to work, or how can they teach others? Does a lady think it menial to teach her cook to make a new dish? Those who do should be taught "the way to a man's heart is down his throat." I do not find "ladies" any kinder, gentler, or more efficient, than the truly respectable class of girls from whom I draw my probationers. Girls who only take to "nursing" to escape something they dislike at home, to get cured of a love affair, to obtain a husband—for we all know "love drives many in and many out of a hospital" should be made to pay well to the support of hospitals. Those who really have to earn their living should be carefully chosen, tested, and tried; for hospital work is hard and dirty—there is no mistake!—then we shall not have so many complaints from "lady nurses." I think a great deal wants doing for the healthy comfort of the resident staff, many (even provincial hospitals) have no garden, all alike are never out of the smell and sound of wards. My nurses are out every day, have half a-day off once a fortnight, and three weeks' holiday during the year.

### Presentations.

On March 13th, Mrs. D'yer, the much-beloved and respected matron of Darenth Asylum, was presented with a very handsome travelling trunk and literary machine by her nurses, and a beautiful little work-basket by the patients. Mrs. D'yer has been an officer of the Metropolitan Asylums Board for thirteen years, and her retirement, owing to ill-health, is universally regretted.

### Vacancies.

The following vacancies are announced:—

**Matrons.**

Princess Alice Memorial Hospital; £50.

**Superintendents.**

Birmingham Scarlet Fever Horpital. Glasgow Royal Infirmary (Night); £30.

**Nurses.**

Bedford Nurses' Institute; £25.
Denbigh Infirmary; £25.
Deaconesses' House, Chester; £25.
Eastbourne Nursing Institution.
Southport Nurses Home.
West Kensington Nurses Home.
County Nursing Institution, Worcester.
Guildford County Hospital; £18.
Nottingham Nursing Institution; £25.
Barrow in Furness Hospital; £20.
Tewkesbury Hospital; £20.
Brecon Infirmary; £18.
Nurses Home, Leicester.
Hampstead Home Hospital, several
Workhouse Infirmary Association, several.
Levic Paris Institution, several.
St. George's Home for Nurses, Sheffield, several.
Nurses Home, Truro; £25.
Royal Westminster Ophthalmic Hospital.
Walsall Accident Hospital (Night).
St. John's Institute, Southport.
Levich Paris Institution.
Bolton Infirmary.
Tunbridge Wells Hospital,; £25.
Sheffield Public Hospital; £22.
Wimbledon Nursing Institution.
Nursing Institution, 96, 97, 98*a*, Wimpole Street, London, W., Mr. Wilson has several vacancies for thoroughly-trained Nurses.
Croydon Union; £20.
Nurses' Institute, Jersey; £25.
Bridgnorth Union; £25.
Nurses' Home, Newcastle, several.
Swansea and South Wales Nursing Institute; £25.

**District Nurses.**

Camborne, Cornhill.

Corbridge-on-Tyne; £75.

Bolton, £25.

Ealing Dean.

**Parish Nurses.**

Brighton.
Guildford.

**Probationers.**

Walsall Accident Hospital.
Guildford County Hospital; £12.
Hospital for Diseases of the Chest, City Road; £12.
Sherborne Hospital.
Bolton Infirmary.
Tewkesbury Hospital.
Barrow-in-Furness Hospital.

### Notes and Queries.

To Correspondents.—1. Questions may be written on post-cards. 2. Advertisements in disguise are inadmissible. 3. In answering a query please quote the number. 4. If a private answer is desired, a stamped addressed envelope must be forwarded.

**Queries.**

(134) *Domestic Medicine.*—Could any nurse tell me the author and publisher of a handbook of "Domestic Medicine;" it costs 2s. or 2s. 6d., and could easily be carried in the pocket? Also where I can get paper pattern of dual garment?—*Celt.*

(135) *Mackintoshes.*—Can any reader of The Hospital give any suggestions for preserving mackintoshes not in use? Would it be of any use to oil them?—*Doris.*

**Answers.**

(120) *Private Nurse.*—If you only want medical *terms* get "Lewis's Pocket Vocabulary" from the publisher, Mr. H. K. Lewis, 136, Gower Street, W.C., for 2s. 8d., 3s. 6d. from other booksellers. If a descriptive dictionary, "Haydn's Dictionary of Domestic Medicine" (Ward, Lock, and Co.), published at 7s. 6d., can generally be got for about 5s. 9d. It is illustrated, but bulky; over 600 pages 8vo.—*Celt.*

(133) *Melbourne.*—There is a good opening for nurses, but no home that I know of. Why not apply to the Alfred or Women's Hospital?—*Cora.*

